# Social media and health – Is it all good, bad or just ugly?

**DOI:** 10.4102/safp.v64i1.5549

**Published:** 2022-06-10

**Authors:** Indiran Govender

**Affiliations:** 1 Department Family Medicine and Primary Health Care, Faculty of Medicine, Sefako Makgatho Health Sciences University, Pretoria, South Africa

Coronavirus disease 2019 (COVID-19) and the subsequent lockdowns with restrictions to movement and gatherings brought electronic media, including social media of communication, into immense popularity. Social media has quickly become a vital communication tool for information generation, dissemination and personal use. With people confined to indoor spaces with their families to avoid large gatherings of people, there was an increased use of social media and other digital platforms for information. Social media for communication was effective and allowed for the rapid dissemination of health information during this disaster. Social media created opportunities to keep people safe, informed and connected, and it exploded in popularity.^1^ Information about which drugs to use and which to avoid could be rapidly spread, as well as what non-pharmacological strategies to engage in when managing COVID-19. Information about vaccines was also provided to medical practitioners and laypeople. Information about diagnosis and symptoms of COVID-19 made everyone experts in handling this virus. Thus, we got to know about the role of hydroxychloroquine, angiotensin converting enzyme inhibitors (ACEIs), avoidance of non-steroidal anti-inflammatory drugs (NSAIDs), the debate around use of ivermectin for COVID-19 and correct assessment of symptoms of COVID-19.^1,2,3,4^ Diagnostic criteria for COVID-19 and the value of using steroids in managing severe COVID-19 infections. Health professionals could also provide information to the public about what to do if infected and sick.^2^ However, false information and misinformation spread like wildfire. People in influential positions spread misinformation, which people believed. Mostly, this information was spread without the intention to cause harm, but not checking the validity of information before disseminating it inevitably led to harm.

Thus, we may have learned to use Facebook, Twitter, Snapchat, Instagram, Pinterest, TikTok, WhatsApp and other social media platforms to disseminate valuable health information rapidly, and this particularly appealed to the young. Young people and others are most likely to get their news through social media platforms by relying heavily on mobile devices for communication. Even though young people are at less risk of severe disease from COVID-19, they share the collective responsibility to help stop the transmission. Social media has been useful in surveying public attitudes, identifying infodemics, assessing mental health, detecting or predicting COVID-19 cases, analysing government responses to the pandemic and evaluating quality of health information through prevention educational videos for prevention of COVID-19.^4^ Health professionals relied on this form of health knowledge because physically meeting and listening to experts was not possible, and unlike the printed media, social media communicated rapidly,^5^ which was needed in this time of crisis. Following widespread cancellation of national and international medical conferences, the use of Zoom and other platforms for sharing medical information increased during the pandemic.^6^ However, there is a paucity of studies on the application of machine learning on data from COVID-19-related social media and studies documenting real-time surveillance that were developed with data from social media on COVID-19.^4^ These are opportunities for research.

The majority of people who circulated and recirculated false information did so mostly unknowingly, without making sure of the authenticity of the information. There are also mischievous people who intentionally spread misinformation for many reasons, which may include marketing their products or organisations or marketing themselves as experts. One can only hope we have learned our moral duty to check information properly before disseminating it to our social groups. Maybe there should be repercussions to those who pass information without verifying the facts.

There are many people and organisations who were drastically affected because someone passed on misinformation to groups of people. These were influential people who even spread videos and voice notes, which led to people being unable to continue with their work and put their lives at risk. Some high-profile media figures have promoted scepticism about the COVID-19 vaccines that led many people to forgo vaccination and contract severe disease.

**FIGURE 1:**
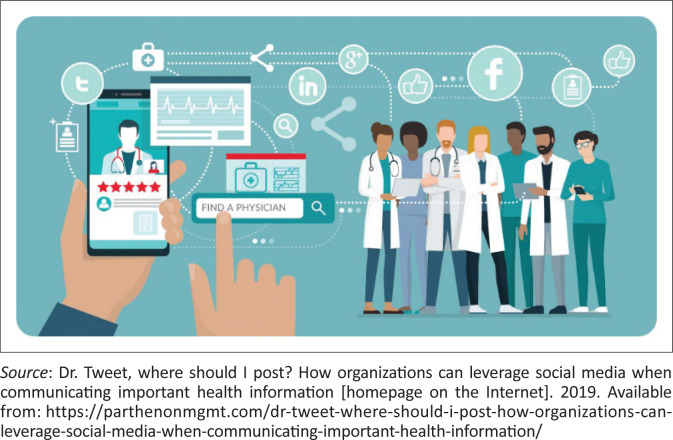
Doctors interact with social media.

Social media as a form of quick communication of vital information and new health knowledge plays an integral role in our lives and is here to stay, but we may need to verify information and put further rules in place to allow for valid and reliable information dissemination (Figure 1)^7^. This may involve allowing for fewer administration people in groups to spread health information and ensure that only correct information that has been verified is sent out. This may take longer than the knee-jerk response of reading something sensational then spreading it like wildfire. Calm down, check information and use professional training experience to critique information. We may need more training for medical professionals in this regard. Thus, although social media has opened many doors, we need to be critical and put rules in place for both dissemination of information and the manner in which readers should take this information in. With regard to COVID-19, social media can play a crucial role in disseminating health information and tackling infodemics and misinformation. Healthcare professionals need to be cognisant of their role in social media and the benefits of social media in healthcare.
